# Decreases in overall light level increase the severity of the reverse Pulfrich effect

**DOI:** 10.1167/jov.25.3.7

**Published:** 2025-03-24

**Authors:** Victor Rodriguez-Lopez, Benjamin M. Chin, Johannes Burge

**Affiliations:** 1 Institute of Optics, Spanish National Research Council (IO-CSIC), Madrid, Spain; 2 Department of Psychology, University of Pennsylvania, Philadelphia, PA, USA; 3 Neuroscience Graduate Group, University of Pennsylvania, Philadelphia, PA, USA; 4 Bioengineering Graduate Group, University of Pennsylvania, Philadelphia, PA, USA

**Keywords:** Pulfrich effect, light level, presbyopia, tunable lens, higher-order aberrations

## Abstract

The Pulfrich effect is an illusion characterized by the misperception of the depth and 3D direction of moving objects. Interocular luminance differences cause the classic Pulfrich effect; the darker image is processed more slowly. Interocular blur differences cause the reverse Pulfrich effect; the blurrier image is processed more quickly. A common correction for presbyopia—monovision—intentionally induces the optical conditions that cause the reverse Pulfrich effect. The effect sizes, and the fact that tens of millions of people wear these corrections every day, raise concerns about public safety. However, although the impact of overall light level (e.g., nighttime vs. daytime) on the classic Pulfrich effect has been well characterized, its impact on the reverse Pulfrich effect is unknown. Here, using a custom binocular 4f tunable lens optical system that allows the decoupling of retinal illuminance and retinal blur, we report how the classic and reverse Pulfrich effects scale with overall light level. Both effects increase logarithmically with decreases in light level. These results motivate a characterization of how light level interacts with other optical factors (e.g., higher-order aberrations) that are likely to impact the reverse Pulfrich effect and, hence, the perceptual consequences of monovision corrections.

## Introduction

Catching a frisbee in the hour before night falls is more difficult than during the day. Often, the frisbee arrives before one has had time to react. This phenomenon is explained, in part, by the fact that visual processing is slower when the overall light level is lower. Visual signals that are processed more slowly leave less time for action planning and motor response. The difficulties for vision further increase when the images in the two eyes differ from one another in certain ways. When playing catch, a person often has about 500 milliseconds to see, plan, and catch the thrown object. If signals from the eyes take longer to reach the brain—for example, 150 ms instead of 50 ms—the time to plan and catch the ball is notably reduced. For example, dramatic misperceptions of depth and the three-dimensional direction of motion occur when the image in one eye is, for example, brighter or blurrier than the image in the other eye (Burge & Cormack, 2023; Burge & Cormack, 2024; Burge & Dyer, 2023; Burge, Rodriguez-Lopez, & Dorronsoro, 2019; Lit, 1949; Pulfrich, 1921; Rodriguez-Lopez & Dorronsoro, 2023; Rodriguez-Lopez, Dorronsoro, & Burge, 2020). These illusions are known, respectively, as the classic and reverse Pulfrich effects. Signals from the brighter or blurrier eye are processed more quickly than those from the other eye. Signals from the darker or sharper eye are processed more slowly. For moving objects, the differences in processing speed cause effective neural disparities, resulting in the aforementioned illusions.

Under what circumstances do the images in the two eyes ever differ in retinal illuminance or blur? When the left and right eyes fixate on the same point in a scene, substantial differences in retinal illumination between the left- and right-eye images might occur when viewing specular objects, or when looking at a scene through a pair of sunglasses with one missing lens. Such viewing situations are relatively rare. Substantial blur differences (e.g., ±1.0 D or more), on the other hand, are comparatively common (Lee et al., 2017; Linke, Richard, & Katz, 2011). Anisometropia, a condition characterized by interocular differences in optical power of ±1.0 D or more is thought to occur in up to 30% of some important demographic groups (Abrahamsson & Sjöstrand, 1996; Weale, 2002). And, monovision corrections, which intentionally induce blur differences between the eyes (e.g., 0.75–2.5 D, most commonly around 1.5 D (Evans, 2007)), are being surgically implanted or delivered with contact lenses as popular alternatives to reading glasses, bifocals, and progressive lenses.

Changes in overall light level are commonplace. Over a day, light level changes markedly, ranging from 10^9^ cd/m^2^ during the day to 10^−4^ cd/m^2^ at night (Halsted, 1993; Kunkel, Daly, Miller, & Froehlich, 2016). The main purpose of the present article is to assess how overall light level interacts with image differences between the eyes to determine the temporal characteristics of neural processing in the binocular visual system. Specifically, we ask how overall light level impacts the interocular discrepancy in temporal processing caused by a given luminance or blur difference between the eyes. The answer has implications for basic scientific understanding and for clinical practice in ophthalmology and optometry.

**Figure 1.**
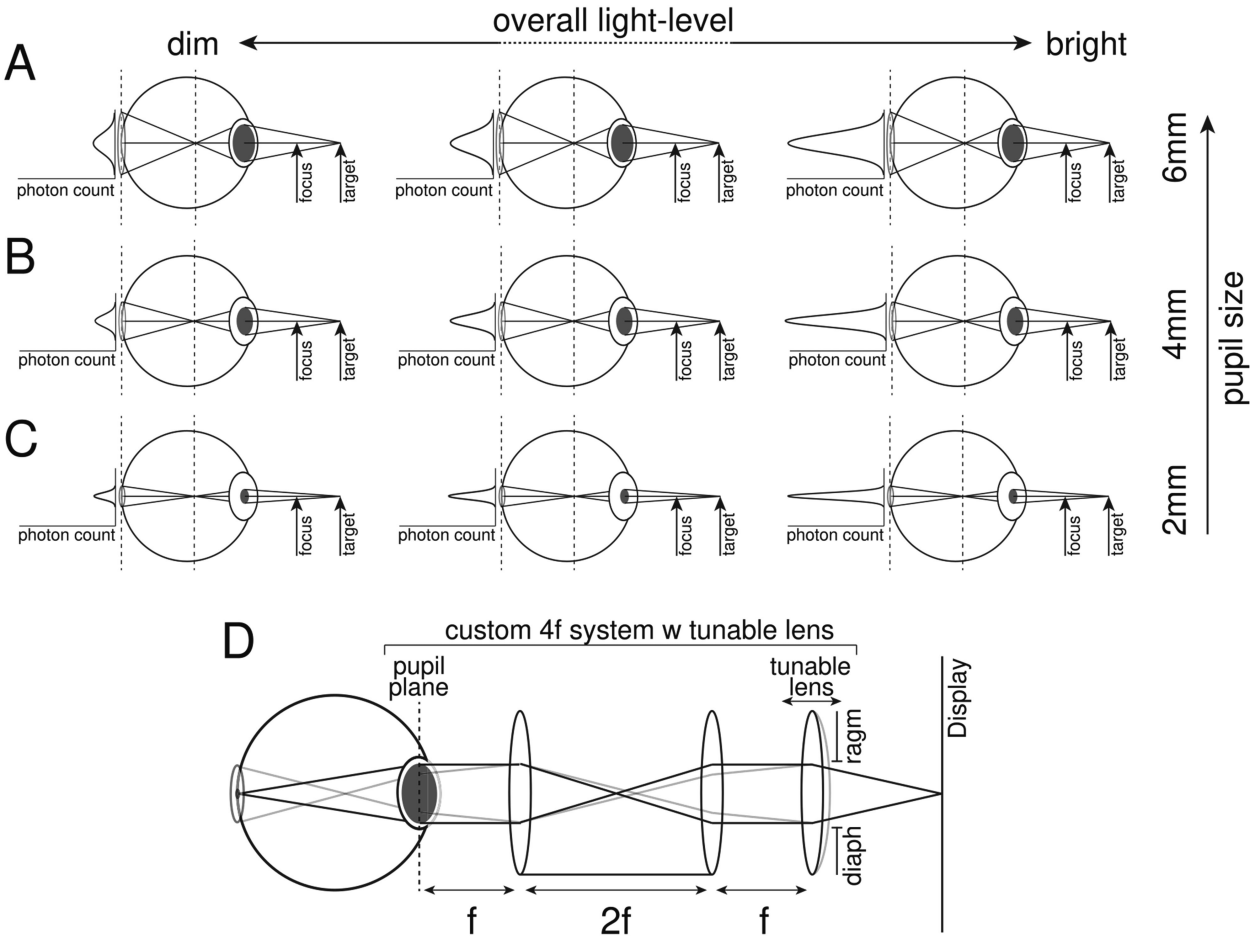
Overall light level, pupil size, retinal illumination, and optical quality. Effect of changing overall light level for a fixed pupil size diameter of 6 mm (**A**), 4 mm (**B**), and 2 mm (**C**). The volume of the photon-count curve represents the amount of light reaching the retina. The width of the curve represents the blur circle. (**D**) Custom 4f optical system used in this study. It projects a tunable lens and a diaphragm into the pupil plane of the eye. The power of the tunable lens is under programmatic control.

Our primary interest is to determine the effect of overall light level on the reverse Pulfrich effect. However, increases in light level cause decreases in pupil size. Smaller pupil sizes reduce the amount of defocus blur for a given focus error, which should reduce the size of the reverse Pulfrich effect. Thus to determine the independent effect of overall light level on the size of the reverse Pulfrich effect for a given level of defocus blur, the ability to fix or monitor the pupil size is required. For these purposes, we used a custom 4f tunable lens optical system. For each combination of overall light level and focus error, we measured human performance in three fixed pupil-size conditions (2mm, 4 mm, 6 mm) and one condition in which the pupil was allowed to vary naturally. Each of the fixed pupil size conditions was enforced by projecting diaphragms of appropriate diameter into the pupil plane of each eye. This aspect of the experimental design allowed us to isolate optical from neural influences on the effects. For the natural pupil size condition, the diaphragm was removed such that the entrance pupil of the entire optical system (4f system + human eye) was determined by the diameter of the human pupil. For both participants, the natural pupil size ranged between 4 and 6 mm for all overall light levels (see Supplementary Figure S1). The 4f optical system included a tunable lens that enabled changes in optical power without inducing magnification differences (Figure 1D). The tunable lens was under programmatic control, allowing us to randomly interleave trials in which the left-eye image was defocused and the right-eye image was sharply focused, and vice versa.

**Figure 2.**
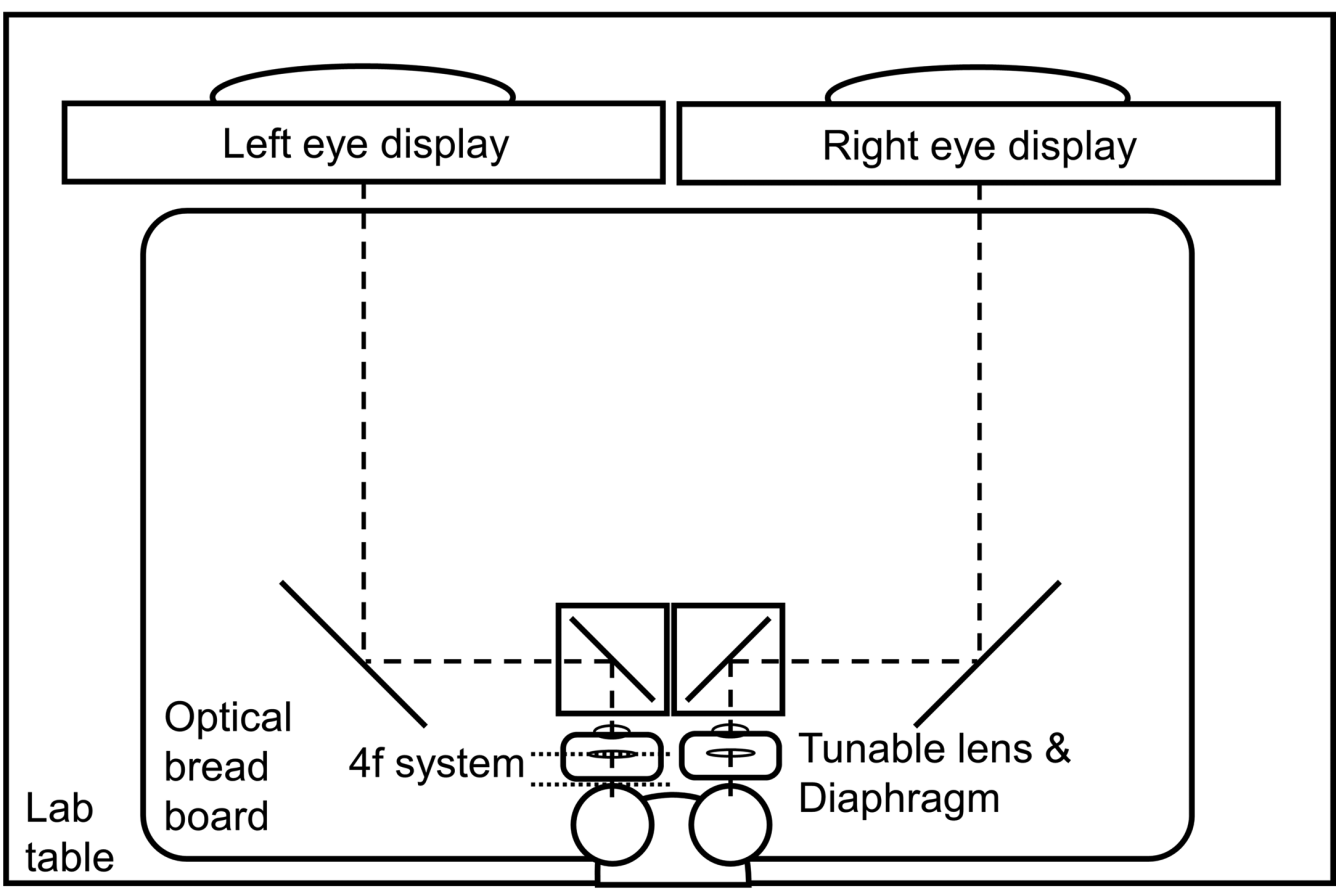
Experimental rig. Representation from above of the optical setup. The 4f optical system shown in Figure 1D is represented as two optical planes. Distances and sizes are not proportional to the real optical system.

Here, we investigate how changes in overall light level change the severity of both the reverse and classic Pulfrich effects. With a haploscope system equipped with a 4f tunable lens system for each eye (Figure 2), we measured the effect of changing overall luminance across nearly two orders of magnitude (∼70×), in each of several fixed pupil size conditions and one natural pupil size condition. The effective luminance of the display accounting for light-loss through the 4f system ranged from 12.8 to 0.3 cd/m^2^. Across the luminance and pupil size conditions, retinal illuminance ranged from 360 to 0.6 trolands. These are values that straddle the borderline between photopic and mesopic light levels. The reverse Pulfrich effect was induced with interocular differences in focus error of 3.0 D^*^, and the classic Pulfrich effect was induced with interocular luminance differences of 75% (see Methods).

Results show that both the reverse and the classic Pulfrich effects increase in strength as overall light level decreases. The effect of light level on the classic Pulfrich effect approximates previous results in the literature (Bernhard, 1940; Lit, 1949; Mansfield & Daugman, 1978; Prestrude, 1971). The effect of overall light level on the reverse Pulfrich effect is novel. Results also show that, for matched retinal illuminance, pupil size has a pronounced effect on the strength of the reverse Pulfrich effect, but it has little effect on the classic Pulfrich effect. Interestingly, however, the effect of pupil size on the strength of the reverse Pulfrich effect does not follow simply from geometric optics. We discuss below these findings and their implications. The effect of light level on the classic Pulfrich effect is similar to previous results in the literature (Bernhard, 1940; Lit, 1949; Mansfield & Daugman, 1978; Prestrude, 1971).

## Methods

### Experimental display rig

We used a custom two-display, four-mirror haploscope system, and a prototype portable 4f optical system to present the experimental stimuli (Figure 2). The displays were two identical VPixx VIEWPixx LED (VPixx Technologies Inc., Saint-Bruno, Canada) displays, with a physical size of 52.2 × 29.1 cm, a spatial resolution of 1920 × 1080 pixels, and a native refresh rate of 120 Hz. To ensure the synchronous presentation of the left- and right-eye images, the displays were daisy-chained together and controlled by the same AMD Radion Pro5300M graphics card with 4GB GDDR6 memory. The displays were gamma-corrected. The light from the displays reached the eyes by first reflecting off a pair of large mirrors and then off a pair of small mirrors. The mirrors were adjusted such that the vergence distance matched the distance of the light path between the monitors and the eyes. The small mirrors were housed in mirror cubes having 2.5 cm diameter circular ports. The mirror cubes were positioned one inter-pupillary distance apart. The effective optical distance of the monitors along the light path from the monitors to the eyes was 80 cm. Thus each monitor pixel subtended 1.58 arcmin of visual angle.

The 4f optical system projected tunable lenses EL-10-30-TC (Optotune, Dietikon, Switzerland) and precision-printed diaphragms of fixed sizes into the pupil planes of the eyes. This system provided a means to programmatically change the interocular focus difference on each trial, and to control the effective pupil size (Figure 1D). A drawback of this system is the substantial loss of light that occurs (∼87%). Through the entire optical system, the maximum luminance was 12.8 cd/m^2^. The optical distance from the eyes of the observer to the screen was 80 cm. Additionally, the baseline power of the tunable lens system was set to 1.25 D to compensate for the distance of the eye to the screen. When the nominal defocus was set to 0.0 D, this baseline power prevented subjects from having to accommodate to clearly focus the display.

As noted, the custom 4f tunable lens systems were portable prototypes. The lens systems were in development for a commercial head-mounted ophthalmic device for simulating multifocal corrections (SimVis Gekko; 2EyesVision, Madrid, Spain). This system was optimized for head-mounted device development and not for conventional psychophysical experimentation on an optical bench. As a consequence, a calibration procedure was required before data was gathered to ensure that the center of optics of each subject's eye and each 4f tunable lens system were properly aligned. This procedure required extensive comparisons of subtle visual markers (vignetting, aspect ratio, tilt, dynamic blur, etc.) and entailed that it was impractical to collect data from naïve observers (see Discussion).

### Stimuli

**Figure 3.**
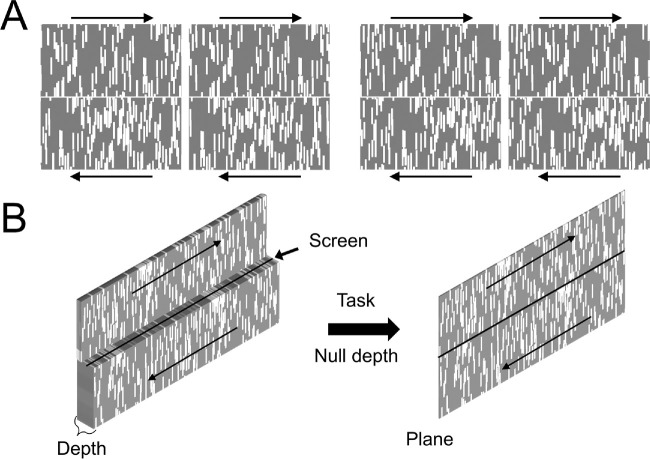
Stimulus and the depth nulling task. Adjacent strips moved in opposite directions. The direction of moving strips was randomly assigned in each run. (**A**) Head on view. On the left, left- and right-eye half-images of a stimulus with a disparity-specified depth step. Free-fuse to see the disparity-specified depth. If the two stimuli are divergently fused, the left stimulus is the left-eye stimulus and the right stimulus is the right-eye stimulus. If the two stimuli are cross-fused, the left stimulus is the right-eye stimulus and the right stimulus is the left-eye stimulus. Divergent-fusing will make the top strip appear farther than the bottom strip. Cross-fusing will make the top strip appear closer than the bottom strip. On the right, left- and right-eye half-images of a stimulus with zero disparity between the images that specifies a flat plane. (**B**) Perspective view. On the left, the upper strip is perceived to be behind the screen, and the bottom strip is perceived in front of the screen. On the right, both the upper and lower strips are perceived to be in the plane of the screen.

The stimulus consisted of four strips textured with randomly positioned 0.25° × 1.00° white bars moving horizontally at a constant speed of 4.0° per second over a gray background. Adjacent strips moved in opposite directions (Figure 3). The number of bars was 375 in each strip. The average overall contrast of the image estimated as the standard deviation divided by the mean of the strip was 0.3.

To induce an effective onscreen interocular temporal shift Δ*t* (i.e., delay or advance), we first determine the equivalent onscreen spatial disparity in degrees of visual angle

(1) $$\begin{array}{c} \Delta x=v\Delta t\; \end{array}$$

where *v* is the movement speed in degrees per second. The onscreen horizontal left- and right-eye positions of the strips in the two eyes evolve with time according to

$$\begin{array}{c} x_{L}\left(t\right)=vt+\Delta x/2\; \end{array}$$

(2) $$\begin{array}{c} x_{R}\left(t\right)=vt-\Delta x/2\; \end{array}$$

where *x_L_* and *x_R_* are the left- and right-eye x-positions in degrees of visual angle, and *t* is time.

When the onscreen interocular temporal shift equals zero, the strips are specified by onscreen spatial disparity to move in the plane of the screen. When the onscreen interocular temporal shift is non-zero, the strips are specified by onscreen spatial disparity to be in front or behind the screen. Negative interocular temporal shifts indicate that the left-eye onscreen image is delayed relative to the right-eye image. Positive interocular temporal shifts indicate that the left-eye onscreen image is advanced relative to the right-eye image. For negative temporal shifts, rightward and leftward moving strips are specified by disparity to be farther than and nearer than the screen plane, respectively. For positive temporal shifts, rightward and leftward moving strips are specified by disparity to be nearer than and farther than the screen plane, respectively.

### Procedure

The task was to set the effective onscreen temporal shift (i.e., the onscreen spatial disparity) via an adjustment procedure until all strips appeared to move in the plane of the screen (Figure 3). In each condition, six runs were conducted. On a given run, the initial onscreen interocular temporal shifts ranged from −15 ms to 15 ms. On a given trial within a run, the strips moved continuously with a fixed temporal shift until the observer made either a coarse adjustment (±1.0 ms) or a fine adjustment (±0.2 ms) indicated with a button press. Each adjustment initiated the next trial, with a new onscreen temporal shift. The observer continued running trials and adjusting the onscreen temporal shift until the observer indicated with a button press that the task had been completed. Throughout each trial, the observer fixated on the rightward-moving strip nearest the center of the screen. Sometimes, the rightward moving strip was just above the vertical midpoint of the screen; sometimes it was just below the vertical midpoint. The stimulus texture was updated with a new texture (different random configuration of the bars within the moving strips of the stimulus) in every run.

The critical onscreen delay for a given condition was estimated by averaging the final settings of the onscreen delay across the six runs. The critical onscreen delay indicates the onscreen delay required to null the neural delay caused by a particular interocular difference in defocus blur or retinal illuminance. When the critical onscreen delay is negative, the left-eye image has to be delayed onscreen relative to the right-eye image for the bars to appear to lie in the same depth plane. When the critical onscreen delay is positive, the left-eye image has to be advanced onscreen relative to the right-eye image. The standard deviation of the final settings indicates the uncertainty with which the final settings were set. It is clear from a cursory examination of the data that the effect sizes are much larger than measurement uncertainty.

### Overall light level

To compute the overall light level in the context of the current experiments, the luminance output of the monitors, the light loss along the optical path to the eyes, and the effective pupil size must all be determined. First, we measured the maximum luminance output of the monitors using a spectroradiometer (120 cd/m^2^). Next, we measured the effective luminance of the monitors through the custom 4f system and verified that the measurements agreed with calculations indicating the expected light loss. Light loss due to the custom 4f system was high (∼87%), so the maximum effective monitor luminance was 12.8 cd/m^2^.

Stimuli were presented at four overall luminance levels ranging from 12.8 cd/m^2^ (maximum luminance reaching the eye) to 0.2 cd/m^2^. To reduce the luminance from the maximum level, we positioned a neutral density filter into the light path for each eye. The neutral density filters were always matched between the eyes and had optical densities (OD) with one of four values (0.0, 0.6, 1.3, and 1.8). These optical densities correspond to transmittances of 100%, 25%, 5%, and 1.5%; the three darkest overall light levels correspond to 4×, 20×, and 67× less light than the brightest condition.

### Pupil size

Pupil size was controlled by projecting a diaphragm of known size into the pupil plane of each observer using the custom 4f-optical system referenced above (Figure 2). The pupil was dilated to ensure that the entrance pupil diameter was determined by the projected diaphragm. Measurements were taken with fixed pupil diameters of 2 mm, 4 mm, and 6 mm. Measurements were also taken with natural pupil diameter. For the natural pupil conditions, a diaphragm with a 10 mm diameter was inserted into the 4f-optical system such that the entrance pupil of the optical system was determined by the natural pupil of the observer's eye.

To obtain measurements of the natural pupil size, post hoc measurements were taken under the exact same stimulus and lighting conditions as the main experiment. Observers wore Pupil Core 120 Hz camera glasses (Pupil Labs, Berlin, Germany), a mobile eye-tracking system that also provides pupillometry. Pupil diameter data were obtained for one second (i.e., 120 frames) after three seconds of adaptation to the light level. We report the average across frames as the pupil size for a given stimulus condition. The results indicated that the natural pupil ranged in size from approximately 4 mm to approximately 6 mm, from the highest to the lowest overall light level (see Supplementary Figure S1).

### Experiments

The conditions were grouped into two different experiments. The first measured the reverse Pulfrich effect. The second measured the classic Pulfrich effect.

The reverse Pulfrich effect was measured by inducing focus error in one eye while leaving the other eye unperturbed. The interocular blur difference in diopters is given by the difference between the focus error induced in the right eye minus the focus error in the left eye:

(3) $$\begin{array}{c} \Delta F=\;F_{R}-F_{L}\; \end{array}$$

For each overall light level, we measured two conditions. In one, the left eye was defocus blurred and the right eye was sharp (Δ*F* = −3.0 D). On the other, the right eye was defocus blurred and the left eye was sharp (Δ*F* = 3.0 D). These defocus differences were induced by increasing the power of the tunable lens in front of either the left eye or the right eye, thereby setting the optical distance of the corresponding monitor beyond infinity and causing retinal defocus blur. The retinal defocus blur difference, assuming geometric optics, can be calculated from the diopters of defocus and the pupil size as follows:

(4) $$\begin{array}{c} \theta_{b}=A\left|\Delta F\right|\; \end{array}$$

where θ_*b*_ is the blur diameter in radians, *A* is the pupil aperture (diameter) in meters, and |Δ*F*| is the interocular difference in defocus. (See Supplementary Material for derivation of Equation 4.)

The classic Pulfrich effect was measured by reducing the luminance of one eye's image onscreen while leaving the other eye unperturbed. The interocular luminance difference, expressed in units of optical density is given by the difference between the effective optical density associated with the right-eye image minus the effective optical density on the left-eye image:

(5) $$\begin{array}{c} \Delta O=OD_{R}-OD_{L}\; \end{array}$$

For each overall light level, we measured two conditions. In one the left-eye image had lower luminance than the right (Δ*O* = −0.6 OD). In the other condition, the right eye image had a lower luminance than the left (Δ*O* = 0.6 OD). These conditions correspond to the left eye receiving 25% of the light that the right eye receives, and vice versa.

The retinal illuminance for each overall luminance level and pupil size was computed by multiplying the luminance by the pupil area expressed in mm^2^:

(6) $$\begin{array}{c} I=L\cdot \pi {\left(A\times 1000/2\right)}^{2}\; \end{array}$$

where *I* is the retinal illuminance in trolands, *L* is the luminance level in cd/m^2^, and, again, *A* is the pupil aperture (diameter) in meters.

The estimated delay at each retinal illuminance level was calculated by taking the average of the absolute values of the final adjustment settings across the two conditions. The standard error of the estimated delay was calculated by taking the standard deviation of the absolute value of the final adjustment settings and dividing by the square root of the number of settings.

To fit how the onscreen delay changed with retinal illuminance, we used a power function

(7) $$\begin{array}{c} \Delta t=b\cdot I^{m}\;\; \end{array}$$

where Δ*t* is the delay, *I* is the retinal illuminance, and *m* and *b* are the parameters of the power law function that are adjusted to minimize the square error of the fit. Taking the natural logarithm of both sides of the equation shows that, in log-space, the power law function has the equation of a line:

(8) $$\begin{array}{c} \ln\Delta t=m\cdot \ln I+\ln b\; \end{array}$$

We transformed the data by taking the natural logarithm of both onscreen delays and the retinal illuminances and then fit the logged data with least squares regression, weighted by the standard deviation of the final adjustment settings. Performing the fit in log-space was justified because the standard error of the final interocular delay settings across runs was more nearly constant on a log scale than on a linear scale.

Each human observer ran in a total of 64 conditions (i.e., 4 overall light levels × 4 pupil sizes × 2 retinal illuminance differences × 2 defocus differences). Overall, 16 retinal illuminance levels ranging from between 0.6 to 360 trolands were measured. The range of light levels varied between photopic and mesopic conditions. The whole experiment took approximately three hours to complete. The experimental protocols were approved by the Institutional Review Board at the University of Pennsylvania and were in compliance with the Declaration of Helsinki. All participants provided written informed consent.

Measurements were performed on the same day. First, the natural pupil conditions, and then the 6 mm, 4 mm, and 2 mm pupil conditions. The different luminance, classic, and reverse Pulfrich effect conditions were randomized for each pupil size. Subjects rested for five to ten minutes between pupil sizes.

### Subjects

Subject 1 was 41 years old and emmetropic without the need for optical prescription. Subject 2 was 27 years old and myopic with appropriate optometric corrections (spherical equivalent of −5.00 D in both eyes). Subject 3 was 23 years old and myopic with appropriate optometric correction (spherical equivalent of −2.00 D in both eyes). For all subjects, visual acuity was better than −0.1 logMAR visual acuity with their best optical correction, stereoacuity was lower (better) than 30″ and had no known visual abnormalities.

## Results

We measured the impact of overall light level on temporal processing in the visual system by taking advantage of both the reverse and classic Pulfrich effects. Two experiments were run. The first experiment assessed how changes in overall light level changed the strength of the reverse Pulfrich effect, which is induced by interocular differences in focus error. The second experiment assessed how changes in overall light level changed the strength of the classic Pulfrich effect, which is induced by interocular differences in the amount of light entering each eye. In both experiments, pupil size was either fixed to one of three diameters (2 mm, 4 mm, and 6 mm), or it was measured as it varied naturally during the experiments (see Methods). Across the four overall light levels (i.e., display luminances) used in the experiment, the natural pupil sizes ranged from between 4 mm to 6 mm (see Supplementary Figure S1). In total, there were 16 distinct retinal illuminance levels ranging from between 0.6 to 360 trolands.

Subjects viewed four horizontally drifting strips textured with vertical bars, that were stereoscopically specified to be in front of, in line with, or behind the plane of the screen. The task, in an adjustment procedure, was to adjust the apparent depth until all strips appeared to be moving in the plane of the screen. Neighboring strips always drifted at the same speed in opposite directions (left vs. right), and the onscreen disparity associated with adjacent strips was always equal in magnitude and opposite in sign. The task was intuitive and easy for subjects to perform.

In the reverse Pulfrich experiment, for each overall light level, data was collected in each of the two conditions. In one condition, the left-eye image was more defocused (Δ*F* = −3.0 D) and, hence, blurrier than the right-eye image; in the other condition, the right-eye image was more defocused than the left-eye image (Δ*F* = 3.0 D).

**Figure 4.**
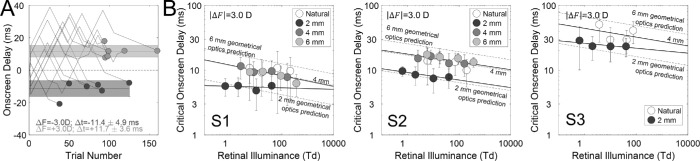
Reverse Pulfrich effect results. (**A**) Onscreen delays presented to a subject during each of six adjustment runs in two separate conditions (jagged lines). The task was to adjust the onscreen delay until the strips appeared to move in the plane on the display (dots). Each run had a different random starting point between −15 ms and 15 ms. Negative onscreen delays indicate that the left-eye image was presented later on-screen than the right-eye image. Positive onscreen delays indicate left-eye image was presented earlier on-screen. Runs from the condition in which the left-eye image was blurred by +3.0 D of focus error and the right-eye image is sharply focused are in dark gray (ΔF = −3.0 D). Runs from the condition in which the left-eye image was sharply focused, and the right-eye image was blurred by +3.0 D of focus error are in light gray (ΔF = +3.0 D). The dashed dark gray and light gray lines represent the average of the final settings in each condition. The shaded dark gray and light gray regions represent the standard deviation of the final settings in each condition. (**B**) Critical onscreen delay for all combinations of retinal illuminance level (abscissa) and pupil size (colors) for subject 1 (left subplot), subject 2 (middle subplot), and subject 3 (right subplot). Critical onscreen delay increases when the retinal illuminance decreases. For the same focus error, the smallest pupil size (2 mm) produced notably smaller effects than other pupil sizes. The reverse Pulfrich effect is stronger when light level is lower. Dashed lines represent geometric-optics-based predictions of the delays in the 2 mm and 6 mm conditions, using the 4 mm condition as baseline.

Figure 4A shows the onscreen delay for the 12 adjustment runs (6 runs × 2 conditions) from one observer at a particular overall light level and pupil size (0.2 cd/m^2^ and 4 mm). (This data is representative of the raw data in other conditions.) The average across the final settings of all six runs in a given condition provides an estimate of the critical onscreen delay that was required to make the drifting bars appear to move in the plane of the screen. This critical onscreen delay should be equal in magnitude and opposite in sign to the neural delay caused by the interocular difference in light level. When the left eye was blurry, the critical onscreen delay was −11.4 ± 4.9 ms (ΔF = −3.0 D). When the right eye was blurry, the critical onscreen delay was 11.7 ± 3.6 ms (ΔF = 3.0 D). These results indicate that the image in the manipulated (blurrier) eye was neurally processed more quickly. These results are consistent with previously reported results (Burge et al., 2019; Rodriguez-Lopez et al., 2020). Thus, blurring an image speeds up how quickly that image is processed by the visual system.

Figure 4B shows the impact of overall light level on the strength of the reverse Pulfrich effect, one panel for each observer. Each data point is the (average) magnitude of the critical onscreen delays in the two defocus conditions at a given light level and pupil size (see Figure 4A). Larger interocular delays are associated with lower light levels, smaller interocular delays are associated with higher light levels, and the change in interocular delay with light level is approximately linear on a log-log scale. For S1 (Figure 4B, left panel), the slope of the linear regression in the log-log space reflects the power of the power function, which is −0.08 ms/td (68% confidence interval = [−0.09, −0.04], which is essentially equivalent to the standard error of the reported statistic) for natural, 4 mm and 6 mm pupil sizes and −0.01 ms/td (68% confidence interval = [−0.05, 0.01]) for 2 mm pupil size. For S2 (Figure 4B, middle panel), the slopes are −0.06 ms/td (68% confidence interval = [−0.09, −0.04]) for natural, 4 mm and 6 mm pupil sizes and −0.06 ms/td (68% confidence interval = [−0.09, −0.03]) for 2 mm pupil size. For S3 (Figure 4B, right panel), the slopes are −0.07 ms/td (68% confidence interval = [−0.21 0.15]) for natural pupil and −0.03 ms/td (68% confidence interval = [−0.07, −0.002]) for 2 mm pupil size.

Note that the interocular delays associated with fixed 2 mm pupils are quite a bit smaller than the interocular delays associated with larger pupil sizes. This is to be expected. A given focus error (e.g., 3.0 D) produces less retinal blur when pupil sizes are small (Equation 4), so the difference in retinal blur between the eyes and, hence, the interocular delay is expected to be smaller.

In fact, theoretical predictions based on geometric optics (Equation 4) and previous empirical results showing a linear relationship between defocus blur and delay (Burge et al., 2019; Rodriguez-Lopez et al., 2020), both predict that delays in the 2 mm pupil-size conditions should be 0.5× the delays in the 4 mm pupil-size conditions. This is precisely what we observe: The prediction (Figure 4B, lower dashed line) aligns with the 2 mm data (Figure 4B, symbols). However, it is a bit surprising that there are no clear differences among interocular delays associated with the 4 mm pupils, 6 mm pupils, and natural pupil sizes which ranged from 4 mm to 6 mm. By the same reasoning as above, the delays in 6 mm pupil size conditions should be 1.5× the delay in the 4 mm pupil-size conditions. However, we did not observe this: The prediction (Figure 4B, upper dashed lines) does not align with the data (Figure 4B, symbols). We speculate that this pattern in the data can be accounted for by the presence of higher-order aberrations in the human eye. We discuss this possibility in the Discussion section below.

In the classic Pulfrich experiment, for each overall light level, there were again two conditions. In one condition, the left-eye image was dimmer and received only 25% of the light that the right eye did (ΔO = −0.6 OD). In the other condition, the right-eye image was dimmer than the left eye (ΔO = 0.6 OD). Six adjustment runs were completed for each condition. (Note that an optical density of 0.6 corresponds to a 25% transmittance, which is equivalent to a 75% light loss).

**Figure 5.**
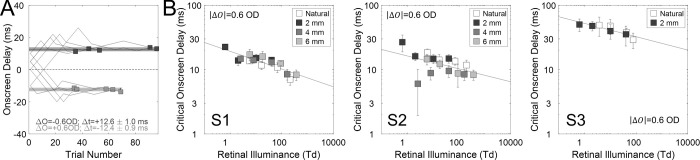
Classic Pulfrich effect results. (**A**) Runs from the condition in which the left-eye image was 75% dimmer than the right-eye image are in dark gray (ΔO = −0.6 OD). Runs from the condition in which the right-eye image was 75% dimmer than the left-eye image are shown in light gray (ΔO = 0.6 OD). In both conditions, both eyes were sharply focused on the screen distance. Runs have different starting points, ranging from −15 ms to 15 ms. A shaded dark or light gray region represents the average and its width the standard deviation across runs, indicating the critical onscreen delay and the uncertainty for both conditions. (**B**) Critical onscreen delay magnitude for every retinal illuminance level (abscissa) and pupil size (colors) for subject 1 (left subplot), subject 2 (middle subplot), and subject 3 (right subplot). Critical onscreen delay increases when the retinal illuminance decreases, and pupil size has no effect. The classic Pulfrich effect is stronger when light level is lower.

Figure 5A shows all 12 adjustment runs (6 runs × 2 conditions) from one observer at another overall light level and pupil size (3.2 cd/m^2^ and 6 mm). This data is representative of the raw data in other conditions. At this light level, when the left eye was dark (ΔO = −0.6 OD) the critical onscreen delay was 12.6 ± 1.0 ms, indicating that the dark left-eye image had to be advanced onscreen to counteract the fact that it was neurally delayed. When the right eye was dark (ΔO = 0.6 OD), the critical onscreen delay was −12.4 ± 0.9 ms, indicating that the left-eye image had to be delayed onscreen to compensate for the fact that the dark right-eye image was neurally delayed (Figure 5A). Unlike in the previous experiment in which the manipulated (blurrier) image was neurally processed more quickly, in this experiment the image in the manipulated (dimmer) eye was neurally processed more slowly.

Figure 5B shows the impact of overall light level on the strength of the classic Pulfrich effect, with one panel for each observer. Clearly, larger interocular delays are associated with lower light levels, and smaller interocular delays are associated with higher light levels. For S1 (Figure 5B, left panel), the slope of the linear regression in the log-log space reflects the power of the power function, which is −0.14 ms/td (68% confidence interval = [−0.16, −0.11]) for all pupil sizes. For S2 (Figure 5B, middle panel) the slope is −0.10 ms/td (68% confidence interval = [−0.15, −0.04]) for all pupil sizes. For S3 (Figure 5B, right subplot) the slope is −0.10 ms/td (68% confidence interval = [−0.13, −0.08]). These results replicate findings reported by Lit (1949) and Prestrude (1971) (see Supplementary Figure S2). Similarly, for S1, the y-intercept of the linear regression in the log-log space is 19.24 ms (68% confidence interval = [17.85, 20.62]). For S2, the y-intercept is 16.3 ms (68% confidence interval = [12.4, 20.06]). For S3, the y-intercept is 52.3 ms (68% confidence interval = [50.13, 56.7]).

Overall light level has a similar impact on the strengths of the reverse and classic Pulfrich effect (see Figures 4 and 5). The most evident difference between the two experiments is that pupil size does not affect the classic Pulfrich effect, whereas it has a systematic effect on the reverse Pulfrich effect. These issues are discussed below.

## Discussion

The classic and the reverse Pulfrich effects both increase in strength as the overall light level decreases. That is, as overall light level decreases, a fixed interocular difference in defocus blur or a fixed (proportional) interocular difference in retinal illuminance causes larger interocular differences in processing speed. The results for the reverse Pulfrich effect are novel. The results reported for the classic Pulfrich effect agree with previously published data.

A notable limitation of this study is its small sample size and the fact that two of the subjects were authors. The custom 4f tunable lens optical systems that we used were portable prototypes that necessitated an alignment procedure that was impractical to perform on naïve observers (see Methods). We note, however, that many articles on related aspects of visual processing, and that are now considered classics, report data from only one or two subjects that were often the authors of the study (Lit, 1949; Lit, 1960; Standing, Dodwell, & Lang, 1968). The facts that, in the present study, the data from each subject resembled the data from the other subjects, that our measurements largely replicate those of Lit (1949) and Prestrude (1971), and that these classic articles have stood the test of time, all suggest the current results are likely to replicate. Nevertheless, to draw more robust conclusions, future research should be conducted on a larger number of subjects with more easily calibrated optical systems.

### The impact of overall light level

Visual processing speed changes systematically with overall light level: interocular delays decrease linearly on a log-log scale which entails that changes in processing speed are well-characterized by a power law. This finding holds true regardless of whether the interocular delays are induced by interocular differences in focus error (Figure 4B) or by interocular differences in luminance (Figure 5B). Changes in the temporal response properties of the retina almost certainly underlie these results.

Evidence indicates that the retina responds more sluggishly when overall light level is lower. This evidence ranges from direct in vitro single-unit recordings (Sinha et al., 2017), to electroretinogram records (Brown, 1968), to a range of tightly controlled psychophysical studies (Drum, 1984; Prestrude, 1971; Standing et al., 1968; Wolpert, Miall, Cumming, & Boniface, 1993). However, although the classic Pulfrich effect has been attributed to retinal processes (Mansfield & Daugman, 1978; Prestrude, 1971; Prestrude & Baker, 1968; Rogers & Anstis, 1972; Wolpert et al., 1993), it seems unlikely that the reverse Pulfrich effect can be attributed to the same physiological site. The classic Pulfrich effect is caused by interocular differences in light level. The reverse Pulfrich effect is caused by different spatial-frequency content in the two eyes (Burge et al., 2019; Chin & Burge, 2022; Min, Reynaud, & Hess, 2020; Rodriguez-Lopez & Dorronsoro, 2023; Rodriguez-Lopez et al., 2020). (The reverse Pulfrich occurs when one eye's image is more sharply focused than the other. The sharper eye is processed more slowly because it contains higher spatial frequencies (Burge et al., 2019)). Neural selectivity for different spatial frequencies does not emerge until early visual cortex (Hubel & Wiesel, 1968). As a consequence, spatial-frequency-based neural differences in processing speed most likely do not emerge until early visual cortex (Levi, Harweth, & Manny, 1979; Nachmias, 1967; Vassilev, Mihaylova, & Bonnet, 2002). Hence, although the primary physiological sites at which the interocular neural delays first emerge that underlie the classic and reverse Pulfrich effects are likely to be retinal and cortical, respectively, the modulatory impact of overall light level on the sizes of these effects can most likely be attributed to light-level-induced changes in the temporal properties of retinal response. Other authors who have studied retinal cells of cats (Dreher, Fukada, & Rodieck, 1976) and of primates (Merigan, Katz, & Maunsell, 1991) have suggested that the magno- and parvo-cellular pathways are responsible for processing spatial and temporal information. In their studies, they measured the function of retinal cells at very specific spatial and temporal frequencies (1 and 2 cycles per degree and 1 and 10 Hz, respectively) and in different regions of the visual field. These studies might imply that the reverse Pulfrich effect could be mediated by either pathway. However, we cannot determine from our data which physiological sites/cellular pathways underlie these effects. Future work could address this question.

### The impact of pupil size

The custom 4f tunable lens system (see Figure 1D) provides precise control over pupil size because it projects a diaphragm of fixed size into the pupil plane of the observer. This aspect of our experimental design allowed us to isolate the modulatory impact of pupil size as overall light level changed.

How pupil size modulates interocular delay is the most striking difference between the results in the two experiments. For the classic Pulfrich effect, with well-focused images, the interocular delay associated with a given proportional difference in retinal illumination was unaffected by pupil size. This is to be expected. Although optical quality changes with pupil size, because both eyes were equally well focused and because pupil sizes were the same in both eyes, changes in optical quality were matched between the eyes. Hence, at a given overall light level, the only factor driving the interocular delays was the proportional differences in light level (|ΔO| = 0.6 OD).

For the reverse Pulfrich effect, the 4 mm, 6 mm, and natural pupil sizes—which ranged between 4 mm and 6 mm—resulted in larger interocular delays than the 2 mm pupil size. This result is expected from elementary geometric optics (see Figure 1 and Equation 4) and from previous findings in the literature regarding the relationship between retinal blur and processing speed (Burge et al., 2019; Rodriguez-Lopez et al., 2020). The reasoning is as follows. Assuming geometric optics and no higher-order aberrations, the amount of retinal blur is linearly related to pupil size for a given focus error (Equation 4). Large pupils yield more defocus blur than small pupils for a given focus error (i.e., 3.0 D in these experiments). Bigger interocular differences in defocus blur cause larger interocular processing delays. Thus a 3.0 D difference in focus error with large pupils is expected to generate larger interocular processing delays than a 3.0 D difference in focus error with small pupils.

The reasoning above accounts for why the 4 mm, 6 mm, and natural pupil sizes resulted in larger interocular delays than the 2 mm pupils. However, it does not account for why delays associated with the 4 mm and 6 mm pupils were so similar to one another (see Figure 4B). Other factors must be responsible. Defocus blur is not the only source of retinal blur in human eyes. Higher-order aberrations also contribute. Importantly, relative to defocus and astigmatism, the impact of higher-order aberrations on retinal blur increases with pupil size (Liang, Grimm, Goelz, & Bille, 1994; Oshika, Klyce, Applegate, Howland, & El Danasoury, 1999; Oshika, Klyce, Applegate, & Howland, 1999; Yan, Kanxing, Ying, Yafei, & Tong, 2003). As a consequence, the presence of higher-order aberrations causes retinal blur to change less dramatically with pupil size for the same amount of defocus than if they were absent. Indeed, the delays for 2 mm pupil size are predicted by geometrical optics, but the delays for 6 mm pupil size are not (Figure 4B). Hence, the similarity of the delays associated with the 4 mm, 6 mm, and natural pupil sizes may be due to higher-order aberrations. Development of a formal model that relates higher-order aberrations to blur discriminability—and hence, processing delay—may be a productive way forward. Proper treatment of this issue would require a very large number of eyes, and is left for future work.

### Monovision, overall light level, and anti-Pulfrich corrections

**Figure 6.**
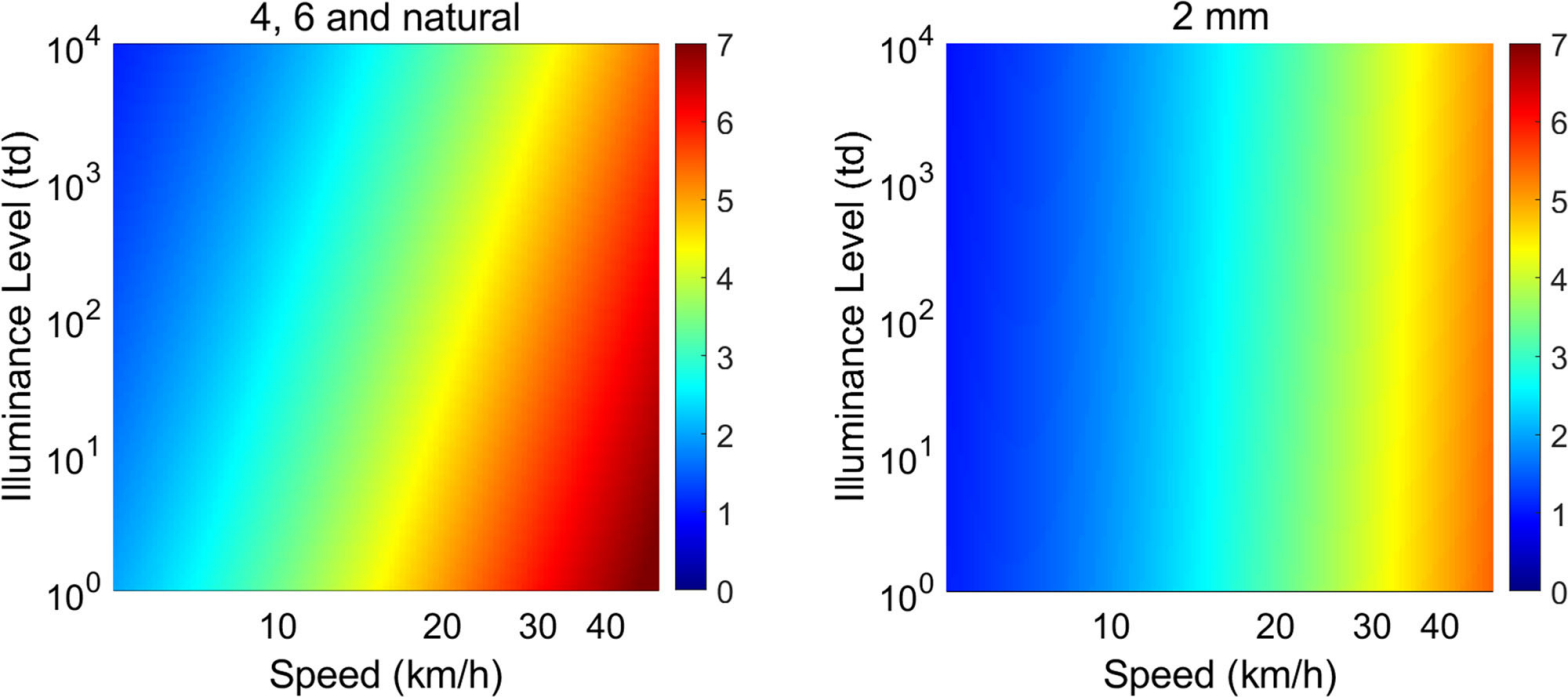
Predicted illusion size in meters of depth misperception for observer S1, assuming a 10 m target distance. Left panel shows the illusion size for the results obtained for 4 mm, 6 mm, and natural pupil sizes. Right panel shows the illusion size for the results obtained for 2 mm pupil size.

Previous research has examined how monovision-correction strengths impact the size of interocular processing delays and the severity of the resultant depth misperceptions caused by the reverse Pulfrich effect (Burge et al., 2019; Rodriguez-Lopez & Dorronsoro, 2023; Rodriguez-Lopez et al., 2020) can be large enough to cause safety concerns. These studies have found that interocular differences in processing speed are proportional to the interocular difference in dioptric power induced by monovision corrections over a wide range. In this study, we showed that decreasing overall light level increases processing delays for a given interocular difference in focus error. In fact, depth misperceptions caused by interocular delays due to differences in optical blur, depending on the distance and the speed of the moving target, and the interpupillary distance can be estimated (see Equations S11–S13 in the Supplementary Material for a more detailed derivation of the illusion size). For example, for S1 (with an interpupillary distance of 65 mm), a target at 10 m distance and moving from 5 to 50 km/h, illuminance levels from 1 to 1000 trolands, and his data for reverse Pulfrich for 4, 6, and natural pupil sizes (slope and y-intercept of the power law are −0.08 ms/td and 11.9 ms, respectively) and for 2 mm (slope and y-intercept of the power law are −0.01 ms/td and 5.6 ms, respectively), Figure 6 shows the predicted depth misperception, associated with a 10 m target distance and variety of retinal illuminances and target speeds. Depth misperception increases with decreasing retinal illuminance and increasing speed, and is about 7 m for the lowest luminance (1 td) and highest speed (50 km/h). Similar calculations can be made for S2 and S3. The findings of this study, as well as this theoretical prediction of the illusion sizes, suggest that monovision corrections may pose an even more significant safety concern under low-light level conditions (e.g., nighttime driving) than at high-light level conditions.

In this study, we used focus differences of 3.0 D between the eyes, which creates optical conditions that are rarely induced by monovision prescriptions. We used this large difference in focus error because pilot data indicated that, of several tested focus-error differences, the 3.0 D difference produced the largest effect sizes (see footnote^*^).

Anti-Pulfrich monovision corrections are aimed at eliminating the depth misperceptions, and hence the safety concerns, caused by the interocular differences in defocus blur induced by regular monovision corrections (Burge et al., 2019). Anti-Pulfrich monovision corrections take advantage of the fact that the classic and reverse Pulfrich effects have opposite signs. That is, darkening an image slows down how fast it is processed; blurring an image speeds up how fast it is processed. At a particular overall light level, the reverse Pulfrich effect that is caused by a given blur difference can thus be eliminated by appropriately tinting the lens of one eye (Burge, Rodriguez-Lopez, & Dorronsoro, 2019; Rodriguez-Lopez, Dorronsoro, & Burge, 2020).

In the current study, we showed that both the reverse and the classic Pulfrich effects increase in strength with decreases in overall light level (except for S2 and pupil size 2 mm). However, these changes in effect size occur at different rates (see slopes in Figures 4B and 5B). More specifically, the classic Pulfrich effect changes approximately twice as fast as the reverse Pulfrich effect with overall light level. Hence, a difference in tint that is effective at one light level would not be effective at another light level. Thus, to develop an effective anti-Pulfrich monovision correction for all light levels, the tint difference would need to change with light level. Photochromic contact lenses (Hammond et al., 2020; Renzi-Hammond et al., 2020) change their transmittance with ambient light level. This technology may enable an appropriate delivery system for an anti-Pulfrich monovision correction that is effective at all overall light levels.

## Conclusions

In this study, we report that the severity of the reverse Pulfrich effect increases when overall light level decreases: the same interocular focus difference causes larger depth misperceptions in nightlight than in daylight. We also replicate multiple studies showing that the severity of the classic Pulfrich is similarly affected. These results motivate a full characterization of how light level interacts with other optical factors (e.g., higher-order aberrations) likely to impact the reverse (and classic) Pulfrich effect(s). Optical technologies like anti-Pulfrich monovision corrections that aim to eliminate the depth misperceptions caused by typical monovision corrections, must account for how the reverse and classic Pulfrich effects change with light level.

## Supplementary Material

Supplement 1

## Footnotes

* We noted above that monovision corrections typically induce focus-error differences of 1.5 D, and rarely exceed differences of 2.5 D. One might have a concern, therefore, that measurements reported here are not reflective of typical monovision prescriptions. We used 3.0 D differences because, in pilot experiments, they produced the largest effect sizes. Pilot data also suggested that interocular delay scales linearly with focus error up to 3.0 D. This informal observation is consistent with published observations that the reverse Pulfrich effect scales linearly with differences in focus error up to 1.5 D (no data on larger focus-error differences has been published). If this latter observation holds, the interocular delay associated with a 3.0 D focus error could simply be divided by two to obtain the interocular delay associated with 1.5 D, the typical monovision correction strength (see Discussion).
