# Supplementary material for: Decreases in overall light level increase the severity of the reverse Pulfrich effect
Source: J Vis. 2025 Mar 24;25(3):7. doi: 10.1167/jov.25.3.7 (PMC11951055; doi:10.1167/jov.25.3.7)
Supplement: Supplement 1 [file jovi-25-3-7_s001.pdf]

SUPPLEMENT

Measurements of pupil size

Supplementary Figure 1 shows the pupil size measured for each overall light-level.

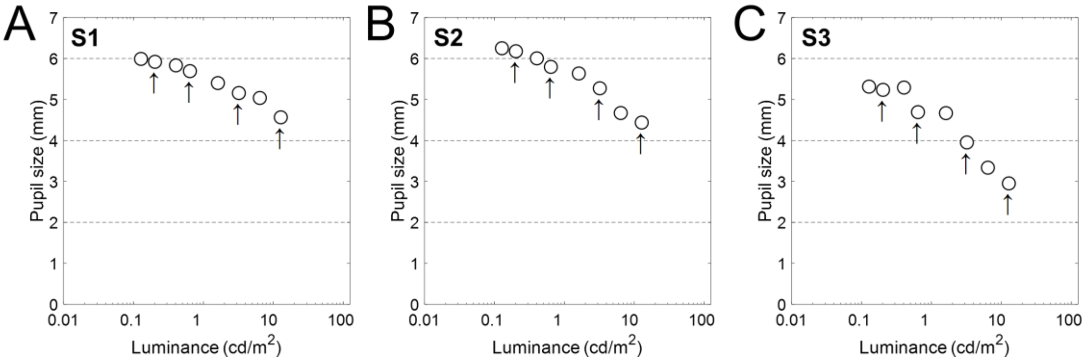

Figure S1. Pupil measurements. Pupil size measured as a function of overall light-level (luminance level emitted by the display) for natural pupil condition. Arrows mark the overall light-level conditions measured in the experiments. Horizontal dashed lines indicate the pupil diameters that were fixed pupil conditions in the experiments (i.e., 2, 4, and 6 mm). **A.** Subject S1. **B.** Subject S2. **C.** Subject S3.

Comparison to classic literature

For the Classic Pulfrich effect, there were data available from other experiments that already performed measurements on how the Classic Pulfrich changes with overall light-level. In both Lit[2] and Prestrude[16], they measured the neural delay for interocular differences in light levels up to $\Delta 0 = 3.0$ OD and for retinal illuminance levels ranging from 2.3 to 2,500 trolands (in our measurements we measure $\Delta 0 = \pm 0.6$ OD and retinal illuminance levels from 0.6 to 360 trolands). Lit measured the perceived depth in distance and later transformed it into a neural temporal delay in two subjects. Prestrude directly measured the neural delay in four subjects. For each luminance level, they measured several interocular luminance differences. However, for interocular differences higher than $\Delta 0 = 1.0$ OD, the neural delay began to behave non-linearly. Besides, we measured luminance differences of $\Delta 0 = 0.6$ OD. For a fair comparison with the methodology of our study, we calculated the neural delay caused by a $\Delta 0 = 0.6$ OD filter for each retinal illuminance level. Then, we estimated the linear regression of the neural delays measured up to $\Delta 0 = 1.0$ OD in their studies. Supplementary Figure S2A and S2B shows these estimations. Supplementary Figure S2C shows the delay as a function of retinal illuminance for both studies from the literature. The slope of the linear regression in the log-log space reflects the power of the power function (see Equation 5), which is -0.23 ms/td for Lit 1949 and -0.24 ms/td for Prestrude 1971.

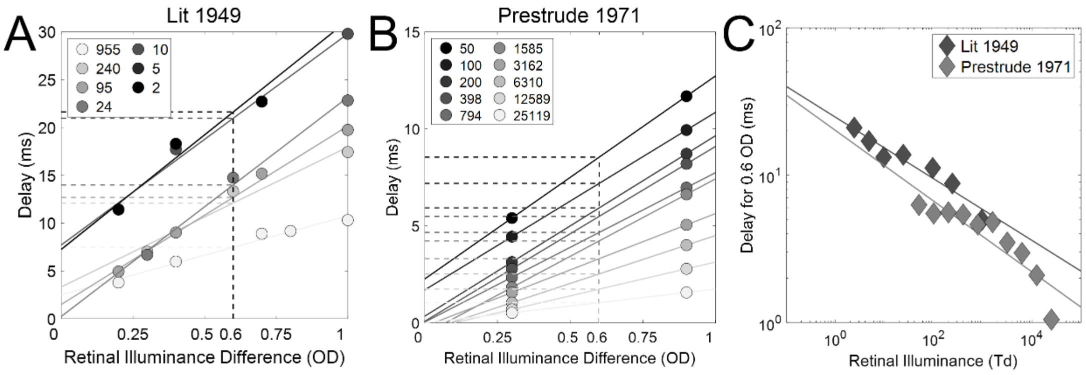

Figure S2. Classic Pulfrich effect for different overall light-levels from the literature. A. Estimation of the delay for $\Delta O = 0.6$ OD from Lit 1949. **B.** Estimation of the delay for $\Delta O = 0.6$ OD from Prestrude 1971. **C.** Delay for a filter of 0.6 OD in ms for every retinal illuminance level measured in Lit 1949 and Prestrude 1971, averaged across subjects.

Geometrical optics: Relationship between blur and aperture

Defocus (i.e., focus error) is defined as the difference in dioptric distance between the focus point and a target point (see Figure S3)

$$\Delta D = D_{focus} - D_{target} \qquad \text{(S1)}$$

where D_{focus} and D_{target} are the dioptric distances to the focus and target points. Diopters are defined as inverse meters, so Equation S1 can be equivalently written

$$\Delta D = \frac{1}{z_0} - \frac{1}{z_1} \qquad \text{(S2)}$$

where z_0 and z_1 are distances to the focus and target points in meters.

The lens equation states that the defocus is equivalently given by the difference in dioptric distance between the imaging plane and the image point

$$\Delta D = \frac{1}{s_0} - \frac{1}{s_1} \qquad \text{(S3)}$$

where s_0 and s_1 are distances to the imaging plane and image point in meters

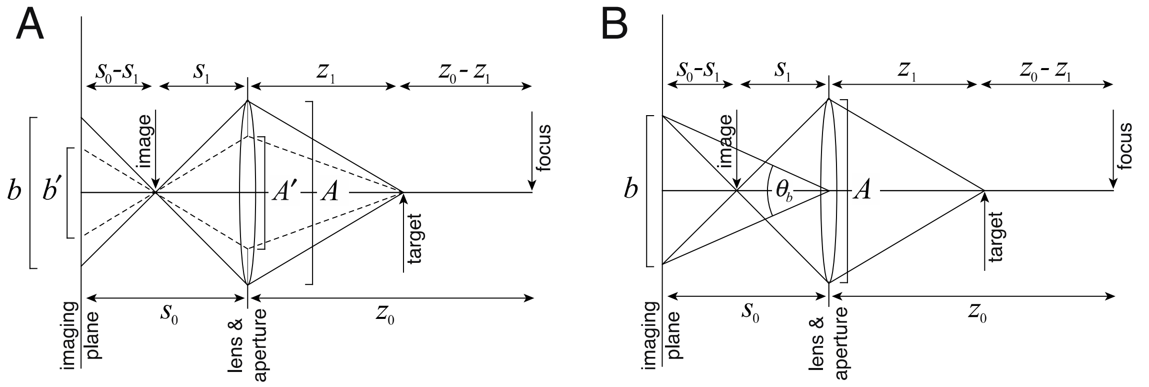

Figure S3. The relationship between focus error, aperture size, blur circle size. A. Blur circle diameter in meters from aperture and defocus. **B.** Blur circle diameter in visual angle.

Using relations between similar triangles

$$\frac{A}{s_1} = -\frac{b}{s_0 - s_1} \qquad \text{(S4)}$$

where A is the aperture diameter and b is the blur circle diameter in meters.

Solving Equation S4 for blur yields

$$b = A\frac{(s_0 - s_1)}{s_1} = A\frac{s_0(s_0 - s_1)}{s_0 s_1} \qquad \text{(S5)}$$

Rearranging Equation S3 yields

$$\Delta D = \frac{(s_1 - s_0)}{s_0 s_1} \qquad \text{(S6)}$$

Multiplying Equation S6 by negative one, taking the absolute value (because the blur circle diameter cannot be negative), and substituting into Equation S5 gives the blur in meters

$$b = A s_0 |\Delta D| \qquad \text{(S7)}$$

The relationship between the blur circle diameter and the subtended visual angle is

$$\frac{b/2}{s_0} = \tan(\theta_b/2) \qquad \text{(S8)}$$

Rearranging after using the small angle approximation

$$b = \theta_b s_0 \qquad \text{(S9)}$$

Substituting Equation S9 into Equation S7 and solving gives the visual angle of the blur circle in radians

$$\theta_b = A|\Delta D| \qquad \text{(S10)}$$

which corresponds to Equation 4 in the main text.

Illusion size estimation

To estimate the illusion size of the depth misperception caused by a processing speed difference between the eyes we used an equation easily derived with basic geometry

$$\hat{d} = \frac{p}{p + v \cdot \Delta t} \cdot d \qquad \text{(S11)}$$

where $\hat{d}$ is the apparent position in depth, p is the interpupillary distance, v is the speed of the target, Δt is the processing speed difference, and d is the actual position. Considering the process speed difference estimated as a function of the retinal illuminance (we recall Equation 7 from the main manuscript) based on experimental data

$$\Delta t = b \cdot \mathrm{I}^m \qquad \text{(S12)}$$

Merging Equations S11 and S12, we can derive the depth misperception as

$$\hat{d} = \frac{p}{p + v \cdot b \cdot \mathrm{I}^m} \cdot d \qquad \text{(S13)}$$

where and m and b are the parameters of the power law function that are adjusted to minimize the square error of the fit.
